# Associations between early motor milestones and speech-language abilities in 4 to 6-year-old children with DLD

**DOI:** 10.1097/MD.0000000000043297

**Published:** 2025-07-11

**Authors:** Nina Stanojević, Saška Žunić, Ružica Bilibajkić, Miško Subotić, Aleksandra Juričić Skevin, Ljiljana Jeličić, Dragana Ćirović

**Affiliations:** a Cognitive Neuroscience Department, Life Activities Advancement Institute, Belgrade, Serbia; b Department of Speech, Language, and Hearing Sciences, Institute for Experimental Phonetics and Speech Pathology ˝Đorđe Kostić˝, Belgrade, Serbia; c Department of Physical Medicine and Rehabilitation, University of Kragujevac, Faculty of Medical Sciences, Kragujevac, Serbia; d University of Belgrade, Faculty of Medicine, Belgrade, Serbia; e Physical Medicine and Rehabilitation Department, University Children’s Hospital, Belgrade, Serbia.

**Keywords:** developmental language disorder, early motor milestones, expressive vocabulary

## Abstract

Developmental language disorder (DLD) is a neurodevelopmental disorder that affects speech and language abilities. However, studies have shown that this disorder is widely connected to other aspects of development. We conducted an observational study on the relationship between early motor milestones and speech-language development in 4 to 6-year-old children with DLD. We studied 30 children diagnosed with DLD and 30 typically developed children. To assess early motor milestones, parents provided written responses regarding their children’s age at which they reached crawling and walking milestones. Speech-language abilities were assessed using the Peabody Picture Vocabulary Test (Third Edition, Croatian adaptation), a measure of receptive vocabulary, Token Test for language comprehension, Vocabulary Test for expressive vocabulary, and Global Articulation Test for articulation. Our results showed that the model in which the timing of reaching crawling and walking predicted results on the Vocabulary Test had significant predictive power; 22% of the variance in expressive vocabulary could be explained by this model (*P* = .03, adjusted *R*² = .229). In diagnosing and planning treatment for children with DLD, the timing of early motor milestones can be considered connected to the future development of expressive vocabulary.

## 1. Introduction

Developmental language disorder (DLD) is a neurodevelopmental disorder characterized by abnormal language development that cannot be explained by mental or physical handicap, hearing loss, emotional disorder, or environmental deprivation.^[1]^ Approximately 7% of the general population has DLD.^[2]^ Within the Diagnostic and Statistical Manual of Mental Disorders,^[3]^ language deficits that mark this disorder manifest as a disturbance in the adoption and use of different language modalities due to deficits in language comprehension and production that involve poor vocabulary, restricted sentence structure, impaired discourse, and general language abilities that are below chronological age expectations and lead to impairment of effective communication, social participation, and academic and professional success. Within the International Classification of Diseases,^[4]^ the diagnosis of DLD requires that children’s language abilities must be more than 2 standard deviations below average for chronological age and more than 1 standard deviation (SD) below their nonverbal abilities.^[4]^ In Slavic languages, the intricate inflectional and syntactic systems (comprising case, gender, number, tense, aspect, and mood) pose significant challenges for all children during language acquisition.^[5]^ For children with DLD, these complexities represent an even greater cognitive burden.^[6,7]^ The need to master numerous inflectional forms, as well as the correct use of function words, places higher demands on their language learning abilities. As a result, children with DLD in these languages may face increased difficulty in acquiring proper sentence structures, verb conjugations, and noun declensions, further complicating the diagnostic process.^[8]^ For example, while typically developing (TD) children can rely on context and word order to infer meaning, children with DLD might struggle with the grammatical markers that carry key information about relationships between elements in a sentence. This additional challenge underscores the importance of developing diagnostic tools and intervention strategies that account for the unique linguistic features of Serbian languages, as standard vocabulary-based assessments may not fully capture the language difficulties experienced by these children.

The development of motor skills in early childhood plays an important role in a child’s language development. Iverson^[9]^ specifically notes that motor skills acquired by 18 months of age have the most significant influence. Other studies examining the impact of motor skills on speech and language development have also confirmed this connection.^[10,11]^ The existing literature reveals that the influence of gross motor skills on language abilities is both immediate and longitudinal, and can be detected during toddlerhood and the preschool years. Research investigating the timing of the emergence of certain gross motor skills (such as sitting or walking) has shown that changes in posture and locomotion can predict both receptive and expressive language abilities, not only at the time of acquisition but also over time.^[10,12–14]^ The connection between early motor abilities and later speech-language abilities in children with typical language development has been the subject of many studies, but the results are contrary, given that some studies showed the connection^[12,15]^ while others did not.^[16,17]^ Some of the reasons for the different results are the different ages at which speech-language measures were applied and the different motor and speech-language aspects that were measured.

Studies examining motor abilities in children with DLD have demonstrated that this population shows distinct patterns in motor skill development.^[18–20]^ Children with DLD have less developed motor skills compared with TD children.^[21–25]^ Differences have been found in the timing of early motor milestone achievement,^[20]^ current motor abilities,^[26]^ and the speed of performing motor tasks.^[19]^ In the study by Diepeveen et al,^[20]^ it was shown that in a group of children with DLD, there were more of those who were late in early motor milestones than in the TD group. They concluded that DLD is a disorder that not only affects brain regions responsible for speech and language, but also broader brain dysfunction. According to Bishop,^[21]^ motoric deficits of children with DLD have a stronger relationship with speech than with language, that is, the ability to produce speech sound correctly is directly related to the motor abilities of an individual. Other researchers who studied the connection between motor and certain receptive and expressive language abilities in children with DLD have shown that there is a correlation between these language abilities and fine or gross motor skills.^[26]^ Thus, current motor abilities have been found to correlate with both speech-language and cognitive skills in children with DLD as well as in TD children.^[26]^.

The relationship between motor and language development is bidirectional. On one hand, certain brain regions are responsible for both motor and language functions simultaneously, such as the premotor and motor areas of the left frontal lobe and the somatosensory areas of the parietal lobe.^[27]^ On the other hand, a connection exists at the behavioral level, as the development of locomotion enables the child to establish new patterns of communication and engage in different forms of interaction with their environment.^[10,12]^ Given that it has been shown that DLD is characterized by unique features not only in language development but also in motor development, investigating the relationship between these 2 domains in this population is of particular interest. The relationship between current motor abilities and speech-language skills has been confirmed in both TD children and those with DLD. However, the relationship between early motor abilities and later speech-language development has been investigated only in TD children,^[15,16,28–30]^ but, to our knowledge not in children with DLD. This raises the question of what the nature of the relationship is between early motor abilities and later speech-language skills in children with DLD.

The potential relationship between early motor milestones and speech-language abilities in children with DLD aged 4 to 6 years was the subject of our study. In the present study, we examined the differences in the time of reaching early motor milestones between children with DLD and TD children, and the association between the time of reaching early motor milestones (time of reaching crawling and walking) and speech-language abilities (receptive vocabulary, expressive vocabulary, language comprehension, and articulation) in children with DLD aged 4 to 6 years.

## 2. Methods

### 2.1. Sample

The study was conducted during 2021 and 2022 in Belgrade, Republic of Serbia. The study sample consisted of 60 participants of both sexes, aged 4 to 6 years. The sample consisted of 2 groups: the first group consisted of participants with DLD, while the second group consisted of participants with typical speech-language development (TD). All children from the DLD group have been recruited from the Institute for Experimental Phonetics and Speech Pathology “Đorđe Kostić” (IEPSP) from Belgrade, where they were diagnosed by a speech-language pathologist with 20 years of experience and admitted for speech-language therapy. Additionally, the diagnosis was confirmed based on the results of the Serbian standardization of the Brine-Lezin scale^[31]^ and the WISC.^[32]^ The inclusion criterion for the DLD group was evidence of language impairment (verbal intelligence quotient score lower than 1.25 SDs below average), according to the widely applied rule in defining children with DLD.^[2,33–35]^ The TD group consisted of 30 participants recruited from a local kindergarten with speech-language development, in accordance with their chronological age. The exclusion criteria for both groups were as follows: performance IQ < 85, presence of neurological damage, pervasive developmental disorder, history of paroxysmal attacks of any kind, premature birth, low birth weight (<2500 g), vision and hearing impairment, use of medications that affect psychomotor functions, and residence outside of Belgrade. An additional exclusion criterion specific to the DLD group was that participants must not have received more than 1 week of speech-language therapy. Two additional inclusion criteria were applied to both groups: (1) parents were required to have more than 12 years of formal education, and (2) a written diary documenting the child’s motor development was required. A developmental diary was defined as a written record of the timing of specific developmental milestones, with particular emphasis on crawling and independent walking.

The DLD and TD groups were balanced based on age. The mean age for the DLD group was 65.70 (SD = 9.80) months and that for the TD group 66.60 (SD = 10.15) months (*P* < .05). Groups were also evenly distributed (*P* > .05) for performance IQ, with the DLD group having a mean score of 101 (SD = 11.27) and the TD group having a mean score of 105.80 (SD = 9.66). There were 22 male participants and 8 female participants in the DLD group, and 18 male participants and 12 female participants in the TD group. A Chi-square test of independence showed no significant difference in gender distribution between groups, *χ*² (1, N = 60) = 1.20, *P* = .27.

### 2.2. Instruments

The data about children’s motor development were gathered based on written parental reports. Parents were asked to report the age (in months) at which their child achieved specific motor milestones, including crawling and independent walking. The questionnaire consisted of 12 questions in total. Parents completed the questionnaire prior to the speech-language assessment during the initial evaluation session.

The short version of the Token Test^[36]^ was used to assess language comprehension abilities. Participants were shown objects of different shapes (circles and rectangles), sizes (small and large), and colors (green, blue, red, yellow, and white). The examiner gives instructions to the participant (“Touch the yellow circle”). Where the test instructions became more complex and longer. The test contained 6 subparts, with 39 items in total. We used pass–fail scoring; thus, the maximum number of points was 39.

The Peabody Picture Vocabulary Test (PPVT-III-HR)^[37]^ was used to assess receptive vocabulary. The children’s vocabulary was assessed by showing 4 pictures per word. The task required the child to point to a picture named by the examiner. The test began with a set of pictures that were estimated to be appropriate for the child’s age. The test ended when the child makes 8 or more errors in the set. The results are presented as standardized scores.

For the assessment of expressive vocabulary, we used the Vocabulary Test for Children aged 3 to 7 years.^[38,39]^ It consists of 5 subtests within which words are arranged according to age. For the age of 3, the test consists of 20 nouns, for the age of 4 consists of 40 nouns, for the age of 5 consists of 60 words (nouns, verbs, and adverbs), for the age of 6 consists of 80 words, and for the age of 7 consists of 100 words to say. All tests were performed individually. Respondents were asked to name the terms shown in the picture. For example, the respondent was shown a picture of a house, and then asked the question, “What is in the picture?” If the answer is a house, then the next question is, “What is a house?” If you get any definition of a house, for example, “living there,” “sleeping,” or “living,” etc the respondent receives 1 point. Half a point on the first question and half a point on the second one. Thus, if we examined children from the age of 3, they could achieve 19 points for specific nouns because the first part of the test consisted of 19 words that represented specific terms. In addition, this part of the test contained 1 abstract noun (“life”). The respondent is asked the question “What is life?” If the respondent gives any answer from which it can be concluded that he has some idea of the meaning of this word, he gets 1 point. Therefore, an examinee between the ages of 3 and 4 can score a total of 20 points. In this way, the answers at older ages were also shortened, with the number of abstract nouns increasing with age.

The Global Articulation Test was used to assess articulation abilities.^[40–42]^ This test evaluates the pronunciation of speech sounds in Serbian. The Serbian language contains 30 sounds; thus, this test consists of 30 pictures that the examinee must name or repeat the name after the examiner. We assessed whether the respondent’s pronunciation of the targeted speech sound was correct or incorrect, and it was considered incorrect if the sound was omitted or replaced by another sound, or if the quality of the sound pronunciation was inadequate. In the present study, we analyzed the number of incorrectly pronounced sounds.

### 2.3. Procedures

Based on documentation from the IEPSP, children diagnosed with DLD who met the defined inclusion and exclusion criteria for the DLD group were identified. Participants were not allowed to have been enrolled in speech-language therapy for more than 1 week prior to inclusion in the study. After that, parents were contacted to collect additional information, specifically whether they possessed a written diary documenting their child’s development. Following this, parents were invited to bring their children for testing and to complete the questionnaire. These procedures were repeated until a total of 30 participants were recruited.

Local kindergarten was contacted, and after obtaining the contact information of parents of children aged 4 to 6 years known to have no developmental difficulties, these parents were approached through phone call and invited to visit the IEPSP, provided they confirmed possession of a written developmental diary. At the IEPSP, the children underwent the same initial evaluation procedure as those in the DLD group. Based on the collected documentation, children who met the inclusion and exclusion criteria for the TD group were then invited to complete testing, and their parents were asked to fill out the questionnaire.

**Figure 1.**
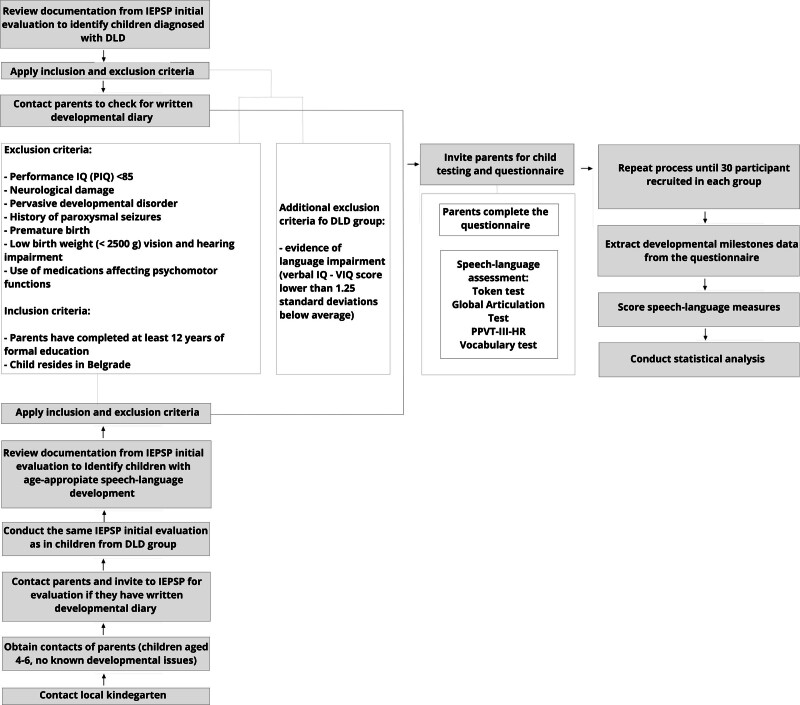
Flowchart of recruitment process and study procedure.

Before the participants’ assessments, parents filled out a report about the child’s data and motor development. The assessment was conducted in 2 sessions over 2 consecutive days. During the first session, the Token test and Global Articulation Test were applied, and during the second session, the PPVT-III-HR and Vocabulary tests were administered. The participants’ parents signed a written informed consent form for their children’s participation in the study. This study was conducted in accordance with the guidelines of the Declaration of Helsinki. The recruitment process and study procedures are presented in Figure 1.

### 2.4. Statistical analysis

Statistical analysis was performed using the IBM SPSS statistical software (SPSS for Windows, version 20.0). The normality of the data distribution was assessed by examining Skewness and Kurtosis. All numerical data that did not fall within the acceptable range of normality were normalized using the rank-case function. All presented mean and standard deviation values are absolute and calculated before application of the function. Differences between groups for all variables were calculated using an independent sample *t* test. Multiple linear regression analysis was used to examine the possible association between independent and dependent variables. The assumption of multicollinearity was assessed using the Tolerance and by variance inflation factor. Each statistic showed a tolerance measure > 0.2, and variance inflation factor < 5; therefore, all results met the assumption for multicollinearity.^[43]^ Statistical significance was defined as *P* ≤ .05. Power analysis was conducted using G*Power version 3.1.9.4 for sample size estimation. Significance criterion was set at α = .05, power = .80, and effect size of 0.50. The minimum sample size needed for linear regression was N = 23.

## 3. Results

The data on group differences in the PPVT-III-HR, Token Test, Vocabulary, and Test of Articulation are provided in the Table S1, Supplemental Digital Content, https://links.lww.com/MD/P376, which includes a table presenting the results for each test. The data on the time of reaching early motor milestones in children from the TD and DLD groups are given in Table 1. The results showed that there was no statistically significant difference between children with DLD and children with TD at the time of reaching crawling (*P* = .52) nor time of reaching walking (*P* = .25).

**Table 1:** Difference between groups in time of reaching early motor milestones given in months.

Group	Crawling mean ± SD	Walking mean ± SD
SLI	8.97 ± 1.25	12.63 ± 1.65
TD	7.73 ± 1.55	12.10 ± 1.32
*T* test	*P* = .52	*P* = .25

Mean = the average value of a set of numerical data, SD = standard deviation.

The data on speech-language measure scores are provided in the Table S1, Supplemental Digital Content, https://links.lww.com/MD/P376. To test the predictive effect of the model using time of reaching crawling and time of reaching waking on the Token Test, PPVT-III-HR, Vocabulary Test, and Global Articulation Test, we applied Multiple Linear Regression. Each model contained the time of reaching crawling and time of reaching walking as independent variables, and one of the speech-language measures as dependent variable. Our results showed a significant predictive power: 22% of the variance of Vocabulary Test results could be explained by this model (*P* = .03, adjusted *R*^2^ = .22). When each variable was observed separately, there was a significant predictive effect of time of reaching crawling on Vocabulary Test (standardized B = -.34, *P* = .01). The other 3 models for the DLD group did not show statistical significance, although there was a statistically significant association between the time of emerging crawling and the token test results (B = -.22, *P* = .03; Table 2).

**Table 2:** Multivariable linear regression results for predictors of speech-language abilities.

Dependent variable	Predictors	B	SE	β	*P*	F value	*P* value
SLI group							
Token Test	Crawling	-.220	.097	-.412	.03*	2.749	.08
	Walking	.001	.078	.002	.99		
PPVT-III-HR	Crawling	.031	.188	.032	.87	.034	.96
	Walking	-.036	.151	-.048	.81		
Vocabulary Test	Crawling	-.343	134	-.449	.01*	4.018	.03*
	Walking	.198	107	.324	.07		
Articulation Test	Crawling	.063	.045	.261	.17	1.952	.16
	Walking	.035	.036	.182	.33		

TD group							
Token Test	Crawling	-.007	.029	-.051	.805	.1446	.86
	Walking	.013	.035	-.075	.717		
PPVT-III-HR	Crawling	.132	.094	.277	.277	1.076	.35
	Walking	-.100	.114	-.172	-.172		
Vocabulary Test	Crawling	-.307	.141	**-.**407	.038*	2.708	.08
	Walking	-.005	.171	-.005	.977		
Articulation Test	Crawling	.058	.025	.435	.027*	2.836	.07
	Walking	-.037	.025	-.227	.234		

B = unstandardized coefficients, β = standardized coefficients, SE = standard error of the coefficient.

* *P* < .05.

## 4. Discussion

In our study, we first examined differences in the time of reaching early motor milestones between children with DLD and TD children, and found no differences in the time of reaching crawling or walking between these 2 groups. We also investigated the association between the time of reaching early motor milestones and speech-language abilities in children with DLD aged 4 to 6 years and found that there is a connection between the time of reaching crawling and later expressive vocabulary at the age of 4 to 6 years in children with DLD. We also found that the time to reach crawling is associated with later language comprehension ability. Our analysis also showed that, for this group of children, the time of reaching crawling can be used as a predictor of expressive language abilities.

Our results regarding differences in the time of reaching early motor milestones between children with DLD and TD children showed that there was no difference in the time of reaching crawling or walking. Previous studies have shown that children with DLD have weaker motor abilities than TD children.^[18,20,26]^ Diepeveen et al^[20]^ found that more children with DLD do not reach gross motor milestones on time in comparison to TD children. They also concluded that these differences are more emphasized when comparing these 2 groups in fine motor milestones. Our results are not contrary, given that in their study, they used motor milestones that are expected at an older age, while we used motor milestones that are expected to emerge until at least 16 months. Other authors who have found that children with DLD have weaker motor abilities than their TD peers have also investigated motor abilities that emerge at a later age.^[25,44–48]^ Our results showed that children with DLD do not differ in early gross motor abilities compared to TD children, but results from previous studies suggest that differences are notable for more complex motor functions, although further longitudinal research is needed to investigate and compare motor abilities over a longer period of time.

In our study, we investigated the connection between the time of reaching early motor milestones and later speech-language abilities in children with DLD aged 4 to 6 years. Our results showed that the time of reaching crawling can be observed as a predictor of expressive vocabulary at ages 4 to 6 in children with DLD. In addition, there was an association between the time of reaching crawling and later language comprehension ability in this group. Early motor and language skills are related at 2 levels. First, there is a neurological connection because motor and non-motor cognitive brain regions have a large number of connections with each other.^[49]^ On the other hand, the connection between early motor milestones and language also exists at the behavioral level because the motor development of infants leads to different prospects of its surroundings, social interactions, object manipulation, and communication.^[13,50]^ Crawling is an important early motor milestone as it impacts the development of sensory-motor systems of the body and motor skill development that occurs later,^[51]^ as well as infants’ perceptions of emotions.^[52]^ This motor skill enables infants to move through space, which facilitates the perception of different objects and enhances communication from adults, consequently leading to more communication and visual-spatial experiences, thus influencing language development. In children with DLD, this influence can be observed even at the age of 4 to 6 years.

Our results showed that there is no connection between walking and speech-language abilities in children with DLD aged 4 to 6 years. There are studies that found a connection between reaching walking ability and language abilities in TD children in terms of those who started to walk showing better expressive and receptive language abilities than those who have not yet emerged this ability. Carina et al^[17]^ investigated time of reaching walking milestone and language abilities at different age, and show that connection exist at age of 2, but that it is not later notable at age of 3. Further research on the connection between the time of reaching walking and speech-language abilities in children with DLD at different ages is needed to better understand this connection.

The development of certain aspects of speech and language is dependent on motor, auditory, somatosensory, and visual processing. In children with DLD, any of these aspects of development may be impaired. Thus, on the one hand, we have several functions that are necessary for the development of different aspects of speech and language. On the other hand, we have the complexity of DLD, where each of these functions can be impaired and thus affect different aspects of speech and language development. The connection of certain motor functions with certain speech-language abilities might not be the same in children with DLD as in TD children because, in addition to motor function, other functions hinder the development of the examined aspects of speech and language. Considering that other authors found various results when examining this relationship in TD children^[13,15,25,50,53]^ it is not surprising that we came to different results in the TD and DLD groups, given that these 2 groups differ both at the level of brain processing and also at the behavioral level. Further research that directly compares these 2 groups is needed in order to draw more conclusive insights regarding the differences in the observed associations. It is important to investigate the connection between these abilities in children with DLD, given that these findings can contribute to the improvement of protocols for prevention, early diagnosis, and treatment planning. Our results contribute to a better understanding of children with DLD, and this knowledge can contribute to the fact that, based on the crawling time, the child’s strengths and weaknesses can be assumed.

A limitation of our research was the observation of only major motor milestones, and a more detailed assessment of motor abilities would provide more precise information about the link between early motor abilities and later speech-language abilities. Another limitation of our study was that it did not account for all potential developmental, environmental, or genetic factors influencing motor and language development in children with DLD. Future research should consider incorporating a broader range of these factors to provide a more comprehensive understanding of the interplay between these domains. A valuable direction for future research would also be the application of a longitudinal design to explore the evolving nature of these connections across various developmental stages, providing a deeper understanding of their trajectory over time. Additionally, future studies would benefit from larger sample sizes, which would enable more robust statistical modeling, including the use of interaction terms to directly compare groups and test whether the strength of associations differs between children with SLI and their typically developing peers.

## 5. Conclusion

An examination of the relationship between early motor milestones and speech-language abilities can contribute to a better understanding of DLD symptomatology in children aged 4 to 6 years, but also to the creation of more precise protocols that would contribute to prevention, early diagnosis, and improved treatment. Crawling is a major motor milestone that influences other developmental aspects, and its connection to future language abilities in children with DLD can be observed at ages 4 to 6. In the process of detection and treatment planning for children with DLD, the connection between the time of reaching crawling and later language abilities should be considered, but further longitudinal studies are needed.

## Author contributions

**Conceptualization:** Nina Stanojević, Ružica Bilibajkić, Ljiljana Jeličić.

**Data curation:** Nina Stanojević, Ružica Bilibajkić.

**Formal analysis:** Nina Stanojević, Ružica Bilibajkić, Miško Subotić.

**Funding acquisition:** Miško Subotić.

**Investigation:** Nina Stanojević, Saška Žunić.

**Methodology:** Nina Stanojević, Saška Žunić, Ljiljana Jeličić, Dragana Ćirović.

**Project administration:** Nina Stanojević.

**Resources:** Nina Stanojević, Ružica Bilibajkić, Aleksandra Jurišić Skevin.

**Software:** Nina Stanojević, Ružica Bilibajkić, Miško Subotić.

**Supervision:** Dragana Ćirović.

**Validation:** Miško Subotić, Aleksandra Jurišić Skevin.

**Visualization:** Nina Stanojević.

**Writing – original draft:** Nina Stanojević, Saška Žunić.

**Writing – review & editing:** Ružica Bilibajkić, Ljiljana Jeličić, Dragana Ćirović.

## Supplementary Material

SUPPLEMENTARY MATERIAL
