# Supplementary material for: Associations between early motor milestones and speech-language abilities in 4 to 6-year-old children with DLD
Source: Medicine (Baltimore). 2025 Jul 11;104(28):e43297. doi: 10.1097/MD.0000000000043297 (PMC12263047; doi:10.1097/MD.0000000000043297)
Supplement: SUPPLEMENTARY MATERIAL [file medi-104-e43297-s001.docx]

Supplement Difference between groups in speech-language measures

| Group | Token test  mean±SD | PPVT-III-HR  mean±SD | Vocabulary test  mean±SD | The Global Articulation  Test  mean±SD |
| --- | --- | --- | --- | --- |
| SLI | 20.43±7.85 | 98.1±13.8 | 37.67±11.19 | 7.43±3.55 |
| TD | 35.9±2.72 | 128.9±9.16 | 59.23±14.45 | 3.17±2.55 |
| T-test | P=.000 | P=.000 | P=.000 | P=.000 |

SD – Standard deviation; mean - The average value of a set of numerical data
